# AnOxPePred: using deep learning for the prediction of antioxidative properties of peptides

**DOI:** 10.1038/s41598-020-78319-w

**Published:** 2020-12-08

**Authors:** Tobias Hegelund Olsen, Betül Yesiltas, Frederikke Isa Marin, Margarita Pertseva, Pedro J. García-Moreno, Simon Gregersen, Michael Toft Overgaard, Charlotte Jacobsen, Ole Lund, Egon Bech Hansen, Paolo Marcatili

**Affiliations:** 1 Department of Health Technology, Technical University of Denmark, Kongens Lyngby, Denmark; 2 National Food Institute, Technical University of Denmark, Kongens Lyngby, Denmark; 3 Department of Chemistry and Bioscience, Aalborg University, Aalborg, Denmark

**Keywords:** Machine learning, Protein function predictions

## Abstract

Dietary antioxidants are an important preservative in food and have been suggested to help in disease prevention. With consumer demands for less synthetic and safer additives in food products, the food industry is searching for antioxidants that can be marketed as natural. Peptides derived from natural proteins show promise, as they are generally regarded as safe and potentially contain other beneficial bioactivities. Antioxidative peptides are usually obtained by testing various peptides derived from hydrolysis of proteins by a selection of proteases. This slow and cumbersome trial-and-error approach to identify antioxidative peptides has increased interest in developing computational approaches for prediction of antioxidant activity and thereby reduce laboratory work. A few antioxidant predictors exist, however, no tool predicting the antioxidative properties of peptides is, to the best of our knowledge, currently available as a web-server. We here present the AnOxPePred tool and web-server (http://services.bioinformatics.dtu.dk/service.php?AnOxPePred-1.0) that uses deep learning to predict the antioxidant properties of peptides. Our model was trained on a curated dataset consisting of experimentally-tested antioxidant and non-antioxidant peptides. For a variety of metrics our method displays a prediction performance better than a k-NN sequence identity-based approach. Furthermore, the developed tool will be a good benchmark for future predictors of antioxidant peptides.

## Introduction

Oxidation is a vital chemical reaction and as such is present in numerous processes both biological and non-biological. One effect from oxidation is the generation of free radicals, a group of molecules containing an unpaired electron, which are often highly reactive and unstable. These molecules can act as oxidants or reductants, by either donating the free electron or pairing it by accepting an electron from another molecule^1,2^. Free radicals, in low concentrations, are essential for several cellular processes, such as protein phosphorylation, activation of transcriptional factors, apoptosis, immunity, and differentiation^3^.

However, high concentrations of free radicals can damage the biological functionality of cells, leading to various diseases by reacting with vital cellular components, such as lipids, carbohydrates, proteins and DNA^3^. The damage incurred by excessive concentration of free radicals is termed oxidative stress^1–3^. Similar complications are seen in food, where spontaneous oxidization of fats, oils, flavouring substances, vitamins and colours can occur when exposed to air in the presence of heat, light, trace metals or already existing free radicals. As a result, undesirable odours, flavours and texture changes, as well as production of unhealthy compounds can occur^4,5^.

Antioxidants are a versatile group of molecules that either directly or indirectly counter the chain reaction initiated by the unpaired free radical electron, thereby reducing oxidative stress. Thus, the addition of antioxidants to food is a powerful approach to diminish food quality deterioration caused from oxidative stress^4^. Antioxidants can be categorized by their mode of action, although individual antioxidants can have more than one^5^.

Antioxidant groups include; (i) free radical scavengers (FRS), molecules that, in low concentrations, inhibit or quench free radicals, thereby delaying or hindering the damage from the free radicals^6^, (ii) chelators, which delay oxidation by forming complexes with metal ions, preventing them from initiating the formation of free radicals^6^, (iii) oxygen scavengers, that likewise delay oxidation by removing oxygen, minimizing deterioration reactions caused by oxygen^7^, and (iv) antioxidant regenerators, that reconstitute antioxidants after they quench free radicals^8^. In this paper we will focus on FRS and chelating peptides, as there is more experimental data available for those groups.

Currently, commercialized antioxidants comprises mainly synthetic molecules^9^. Primarily because synthetic antioxidants are cost-effective and efficient^10^. The disadvantage is their possible toxic and hazardous effects^10^. An increasing tendency among the public to prefer natural rather than synthetic antioxidants has resulted in extensive research to discover such compunds^11^. A potential solution to this is peptides^11–13^. Peptides derived from natural proteins show promise. They are generally regarded as safe and can potentially contain additional bioactive properties (e.g., hypocholesterolemic or antimicrobial)^11^.

The standard approach for discovering antioxidant peptides has been by hydrolysing proteins of interest with a selection of available proteolytic enzymes^12–15^. The resulting hydrolysates are measured for their antioxidant properties, primarily FRS activity, then further purified and analysed by mass spectrometry, to identify the individual peptides containing the antioxidant properties^12^. This trial-and-error approach is , however, both time- and cost-demanding^13^. Insights introduced by computational prediction of peptide antioxidant properties could greatly reduce laboratory work and are therefore highly desirable to develop.

A variety of predictive patterns for antioxidant activity of peptides have been identified in both the sequence order (hydrophobic amino acids such as leucine or valine in the N‐terminal regions of peptides), the individual amino acids [e.g.; sulphur‐containing amino acid residues (cystine and methionine), aromatic amino acid residues (phenylalanine, tryptophan, and tyrosine) and the imidazole ring‐containing histidine] and the secondary structure. Nevertheless, a full understanding of antioxidant properties of peptides is still lacking^6,13,16^. The lack of a defined set of rules makes a theoretical prediction approach difficult. Fortunately, machine learning can be used to circumvent our incomplete knowledge, as a machine learning algorithm can be trained to learn complex underlying patterns from a given dataset and utilize them to predict antioxidant activities^17^.

Previous papers have presented promising predictions for antioxidative properties of small molecules^18^, proteins^19–21^ and peptides^22–24^ using different machine learning algorithms (e.g. Multiple Linear Regression, Support Vector Machines and Random Forest). These models are, as mentioned in a recent review^13^ on the subject, still in their infancy and no current web-server exists for prediction of antioxidant peptides. One obstacle with these standard models is their inability to take amino acid sequences of different lengths as inputs, as their feature vector must be a fixed length^25^. This is usually circumvented by aligning the sequences^26^ or, in cases where aligning is impossible, with feature extraction, i.e. representing the sequences as a feature vector reflecting their properties^22–25,27–29^. Unfortunately, the resulting feature vectors are inherently biased by the method of feature extraction used^30^.

Recent papers^31–33^ have shown the advantages of using deep convolutional neural networks^34^ (CNNs) to evade this bias. A CNN also requires inputs of identical dimension, but its ability to scan and detect patterns in the sequence input removes the need for alignment. For protein sequences of varying length a simple padding, i.e. adding gaps to the end of the shorter sequences until their length corresponds to that of the longest sequence, is sufficient to allow sequences of varying length as input^35^. This will avoid the bias created from subjective feature extraction, as the convolutional layer within a CNN functions as a self-learned feature extraction layer^35^.

Presently, databases with antioxidant peptides are sparse and lack negatives. These shortcomings are crucial as the performance of CNNs (as well as other machine learning algorithms) is linked to the quality and size of its training data. As expanding a dataset is not always possible, various techniques have been developed to mitigate effects from limited datasets. One of them is multi-task learning^36^ where multiple tasks are trained together to exploit their commonalities thereby requiring less data. Especially, the often-encountered problem of lacking negative data can cause problems. Selecting new negatives based on filters has seen some recent success^37^, but the filtering needs to only exclude positives, as the model otherwise learns the rules of the applied filters and not the general rules^38^. Randomly sampling negatives, preferably from a uniform population, is another approach^39^. This avoids any bias when selecting negatives, but has the disadvantage of introducing some mis-labelling^39^.

As peptides can be a valuable source of bioactive molecules, it is of high interest to create a good benchmark dataset of antioxidant peptides and develope a reliable and effective computational method for predicting antioxidant peptides based on their amino acid sequence.

In this work we developed AnOxPePred, a method for predicting both the FRS and chelating properties of peptides. The predicted antioxidant activity is based solely on the peptide amino acid sequence, which contains information about many of its inherent properties (e.g., size, local structure and charge). In the process of constructing this predictor, a curated benchmark dataset, of peptides and their FRS and chelating properties, was created and subsequently used to train a CNN classifier with two output neurons (for FRS and chelating properties respectively). Our tool displayed a better performance when compared to a k-Nearest Neighbours (k-NN) sequence-identity classification approach and is available as a web-server at http://services.bioinformatics.dtu.dk/service.php?AnOxPePred-1.0.

## Methods

### Benchmark dataset

The benchmark dataset (Supplementary Data S1) used in this work was established by extracting data on antioxidant peptides of length 2–30 amino acids both derived from different protein sources (e.g., fish^40^ and dairy^41^) and synthetic^42^, obtained from various published articles and from the BIOPEP-UWM^43^ database. Each peptide was binary labelled for the two classes, free radical scavenger (FRS) and chelator. The classes were labelled 1 (positive) if their source had measured/indicated an activity and otherwise 0 (negative). This extraction resulted in; 696 antioxidant peptides (685 FRS and 81 chelating, 70 of which have both activities) and 218 non-antioxidant experimentally-validated peptides, as seen in Table 1. Furthermore, to diminish homology bias while training, sequences were removed from both the positive and negative peptides so that no pair had more than 90% identity^44^. All sequence identities in this paper were calculated using the Needleman–Wunsch algorithm^45^ with the parameters; 1 for identical, 0 for dissimilar, − 10 for opening and extending gaps and 0 for end gaps.

**Table 1:** Overview over the benchmark dataset.

	FRS	CheL	FRS/CheL	Non-AO	Random	Total
AOdb	615	11	70	218	500	1414
aodb < 90%	606	11	70	217	500	1404

FRS, CHEL, FRS/CHEL and NON-AO are all experimentally-validated peptides obtained from various papers. RANDOM consists of peptides derived from the UniProt^46^ database, with lengths between 2–30 amino acids. AODB < 90% is the number of peptides after removal of sequences, so no pair has more than 90% identity. *FRS* free radical scavenger, *CHEL* chelator, *FRS/CHEL* both FRS and chelator, *NON-AO* non-antioxidant.

Additionally, 500 random peptides with lengths between 2–30 amino acids, with the same length distribution as the positive dataset were extracted from random proteins derived from the UniProt^46^ database. It was ensured that none of these peptides were identical to any peptide in the positive dataset. This amounted to a final, balanced benchmark dataset of 1404 peptides, consisting of 687 FRS and chelators, 717 peptides termed non-antioxidant and a positive to negative ratio of 0.94 and 0.11 for FRS and chelators respectively.

To improve generalization and achieve a robust accuracy of our model’s predictions on unobserved cases, a fivefold nested cross-validation approach was used^29^. The fivefolds were created so that all folds contained similar number of positives and negatives, and FRS and chelators. Furthermore, a upper threshold for peptide identity was enforced, for any two peptides between different folds. Four partitions were made with a threshold of 60, 70, 80 or 90% identity between folds respectively.

### Peptide representation

To enable the peptides as inputs to the model, their amino acids need to be converted into numerical values^47^. This was done using one-hot encoding, which represents each amino acid with a 20 × 1 vector with a single position (corresponding to the specific amino acid) set to one, and all 19 other positions set to zero^48^. Each peptide was therefore represented by a 2D array created by concatenating the 20 × 1 vectors of the amino acids it was composed of. Additionally, each peptide was padded with zero-only vectors of 20 × 1 until reaching the maximum sequence length (30 amino acids) resulting in a 30 × 20 array per peptide.

### Deep neural network architecture

The model was implemented using TensorFlow 1.13 library. The model is composed of an input layer, a convolutional module (Conv1), a fully connected feed-forward module (Ff1) and an output layer. The input layer is the protein sequences in a 3D array (B × 30 × 20) with B depending on the mini-batch size during training and the number of sequences to predict on when testing. Conv1 consists of three parts; a 1D convolutional layer with 128 filters of size 3 × 1 and a stride of 1 followed by a 1D average pooling layer of size 3 × 1 and a stride of 3, and finally a dropout layer with a dropout probability of 10%. Ff1 consists of a fully connected layer with 256 nodes followed by a dropout layer with a dropout probability of 15%. The final output consists of 2 nodes for FRS and chelating activity respectively.

**Figure 1:**
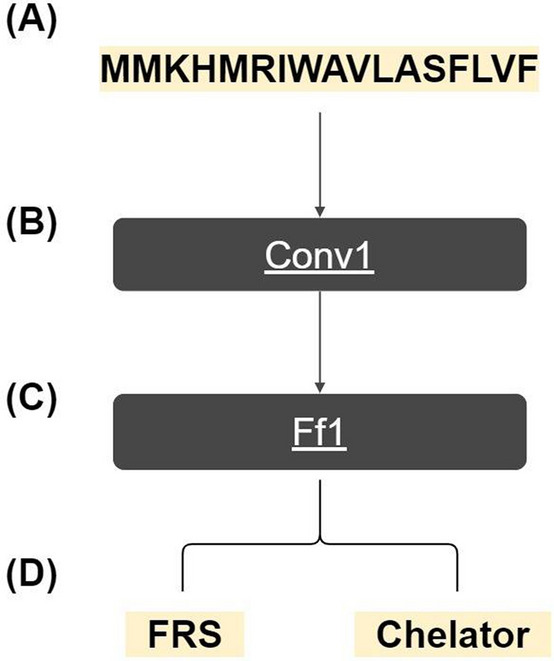
Overview of AnOxPePred’s architecture. Input sequences (**A**) enters the Conv1 module (**B**) which extracts a set of features. The extracted features are then flattened before entering the Ff1 module (**C**). Here the features are used to predict the final output of FRS and chelating properties (**D**). *FRS* free radical scavenger.

The modules were used to construct the model as following. The input layer enters Conv1 which extracts a set of features from the sequences. These features are then flattened (reduced to one dimension) before entering Ff1 and finally from there into the two output nodes as illustrated in Fig. 1. The purpose of Ff1 is to learn which features extracted by Conv1 decides whether a peptide is an antioxidant or not.

The network was optimized with a focal loss^49^ (γ = 3, α = 0.25) and an Adam optimizer (learning rate = 0.00003, decay = 0.005). Exponential Linear Units (ELU)^50^ was used as the activation function for Conv1 and Ff1, and sigmoid on the final output. Dropout was used throughout training as a regularization technique^51^. For training a mini-batch size of 96 was used. All hyperparameters (i.e. nodes, filter sizes, dropout probability etc.) mentioned above were found empirically.

Early stopping was implemented within the fivefold nested cross-validation to reduce overfitting when evaluating the model^52^. The final model used in the web-server was trained on all data for 400 epochs.

### k-Nearest neighbours sequence identity-based benchmark

To evaluate the prediction performance of our model, a sequence identity based k-Nearest Neighbours (k-NN) was designed under the assumption that similar peptides share similar properties. In the same fivefold cross-validation set-up as used for our model, peptides from one fold had their antioxidant activities predicted based on the average annotation of the 5 (k = 5) most similar peptides, in terms of sequence identity, within the four other folds.

### Performance evaluation

To properly evaluate the prediction power for each of the two antioxidant properties (FRS and chelator), the prediction performances were evaluated individually. Area Under a Curve (AUC), F1 score (F1) and Matthew’s Correlation Coefficient (MCC) were calculated for the models (Supplementary Equations S1), as they are metrics commonly used to evaluate classifier performance. AUC values are in the interval of 0–1, with 1 being a perfect agreement, 0 a perfect disagreement and 0.5 implying a random prediction^53^. F1 can be interpreted as a weighted average of the precision and recall, where a score of 1 is the optimal and a score of 0 is the poorest^54^. MCC values are in the interval of − 1 to 1, with 1 being a perfect agreement, − 1 a perfect disagreement and 0 implying a random prediction^55^.

As the predictions from our model and k-NN are continuous values between 0 and 1, a threshold must be defined to change them into binary predictions (0 if below the threshold and 1 if above) thereby enabling the calculation of Recall, Precision, F1 and MCC. This threshold was decided by optimizing for the MCC scores. The final metrics for each model were then the average of the 20 metrics derived from the fivefold cross-validation.

Additionally, the Gini coefficient was used as a measure for how evenly the data was distributed into each fold. The Gini coefficient was derived by calculating the relative mean absolute difference on the number of positive FRS’ in each fold and subsequently dividing it by 2^56^.

### Experimental measurements for radical scavenging activity

Ten peptides were selected from 328.593 peptides derived from proteins studied in the PROVIDE project^57^. These are all proteins that are easily accessible by-products in large-scale industrial processes. The peptides were selected among the ones with the highest predicted FRS scores, with 4 peptides being the highest scoring peptides longer than 15 amino acids. For overlapping peptides, only a single one with the highest predicted score was included in the final set. The final set is reported in Table 2.

**Table 2:** Overview over the 10 new experimentally tested peptide sequences, ranked according to the FRS score predicted by our model (from largest to smallest) and their IC50.

Peptide sequence	Predicted FRS score	IC50 (mg/ml)	
VPFYFEHGPHI	0.64	16.32 ± 1.72	
**HWYD**	0.59	2.24 ± 0.11	
**VWYA**	0.55	7.03 ± 1.25	
**MLWQYKPK**	0.54	6.73 ± 2.42	
EHHNSPGYYDG	0.53	90.83 ± 5.48	
YWTMWK	0.53	14.13 ± 0.93	
ENNRPFAAANEIVPFYFEHGPHIFNS	0.52	38.87 ± 6.53	
LIYPTGCTTCCTGYKGCYYFGKNGKFVCEG	0.52	15.37 ± 5.15	
QSDSDYSSSGPLGVPDPSDLL	0.51	37.00 ± 6.62	
**NNKWVPCLEFETEHGFVYREHH**	0.50	5.47 ± 2.20	
Sodium Casenate		9 ± 2.30	

A low IC50 is evidence for high scavenging activity. Rows are coloured according to their experimental FRS activity (obtained as described in methods) compared to Sodium Caseinate, a known antioxidant. Peptides in bold have a higher activity than Sodium Caseinate, and underlined peptides have a similar (less than twice IC50) activity.

The radical scavenging activity of predicted antioxidant peptides was measured using 1,1-diphenyl-2-picrylhydrazyl (DPPH) radical scavenging activity method of Yang et al.^58^ with some modifications. Peptides were dissolved in dimethyl sulfoxide (DMSO), obtaining peptide solutions in different concentrations (0.0002–0.05 M). Concisely, 100 µL of the peptide solution was mixed with 100 µL of 0.1 mM ethanolic DPPH solution. The mixture was kept in darkness at room temperature for 30 min and absorbance was read at 515 nm. Butylated HydroxyToluene (BHT) solution was included as positive control. Measurements were performed in triplicates. Scavenging effect was calculated as inhibition percentage as in the equation below, where A_s_ is the absorbance of DPPH after reaction with antioxidant peptide, A_0_: Absorbance of peptide solution and ethanol (control), and A_b_ is the absorbance of DMSO and DPPH (blind):

$$ Inhibition\,\,(\% )\,\, = \,\,\left( {1\,\, - \,\,\frac{{A_{s} \,\, - \,\,A_{0} }}{{A_{b} }}} \right)\,\, \times \,\,100. $$

Results were calculated for 50% inhibition concentration (IC_50_) and presented in mg/mL.

As a baseline measure for scavenging activities, we also performed the experiment on sodium caseinate, a common food ingredient with antioxidant properties.

## Results and discussion

### Peptide dataset

**Figure 2:**
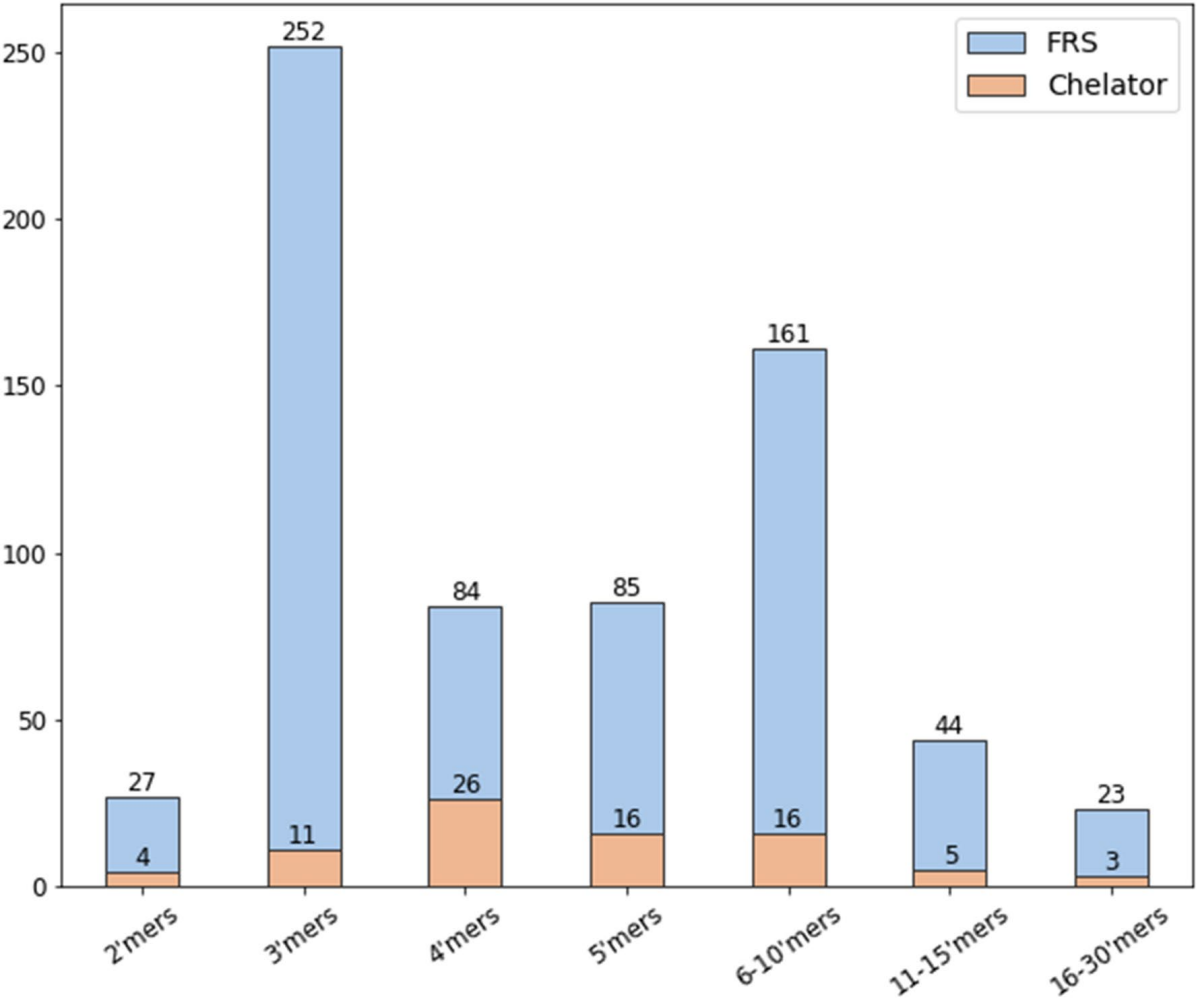
Overview of the properties and length of peptides in the benchmark dataset. *FRS* free radical scavenger.

The antioxidant peptide dataset, constructed as described in the methods section, consists of 687 antioxidant peptides (676 FRS and 81 chelating, 70 of which exhibit both properties) and 217 non-antioxidant experimentally-validated peptides. Peptides were defined as being 2–30 in length. As seen from the peptide length distribution in Fig. 2, a majority of the peptides are short (2–4 mers). A vital component of predicting the activity of underrepresented classes in the training dataset (longer peptides and chelators in this case) is the model’s ability to apply multi-task learning, i.e. exploiting commonalities between short and long and FRS and chelators to improve training where data is lacking.

Additionally, in order to create a more balanced dataset 500 random negatives following the length distribution of the positives were added, achieving a positive to negative ratio of 0.94 and 0.11 for FRS and chelators respectively. Random negatives selection was preferred over negatives retrieved from a filter as there is only a limited understanding of what constitutes an antioxidant, like the seemingly prevalence of certain residues. Random negatives will most likely result in some mis-labelled negatives and a reduced performance but also an unbiased one.

**Figure 3:**
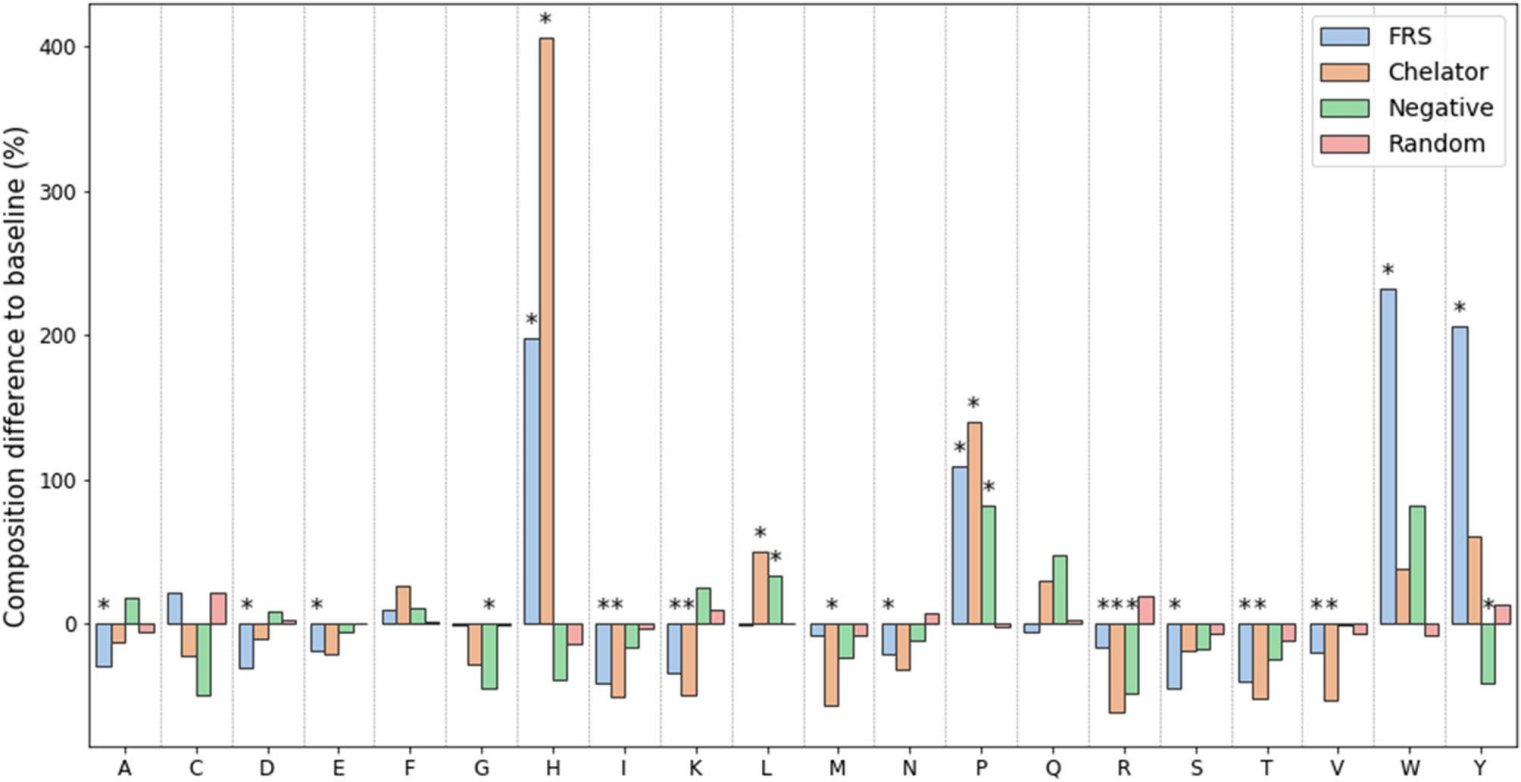
The difference in composition between the antioxidant dataset and a baseline (the average amino acid composition in the UniProtKB/Swiss-Prot data bank). Significant differences (P value < 0.05) was determined by applying a one sample test of proportions^59^ for each amino acid and was marked with an asterisk (*). *FRS* Free Radical Scavenger.

As mentioned, it is expected that certain residues are more prevalent in antioxidant peptides. As an attempt to capture such characteristics, the residue composition of our datasets FRS, chelator, non-antioxidant and randomly selected negative peptides were compared to a baseline composition based on the UniProtKB/Swiss-Prot data bank (Fig. 3), with no restriction to the taxonomic origin of the proteins. Differences in composition was determined by applying a one sample test of proportions^59^ for each amino acid, with an asterisk marking a significant difference (P value < 0.05).

For randomly selected negative peptides no significant difference in amino acid prevalence from the baseline can be seen in Fig. 3, implying they contain no composition bias. Experimentally-tested non-antioxidant peptides have a slight difference in composition, notably a lower ratio of tyrosine, which appears to be connected to antioxidant activity. On the other hand, a more clear residue preference is seen between the composition of antioxidants and the baseline. From Fig. 3, it is evident that histidine is inherently more present in antioxidants, likewise is tryptophan and indeed tyrosine for FRS, supporting their potential relevance for a peptide’s activity. The high frequency of leucine and proline is less intuitive but could be related to the hydrophobic regions that have been observed in antioxidant peptides^6^. Histidine, tryptophan and tyrosine make up a large percentage of the composition in antioxidant peptides, resulting in a number of other residues showing a significant decrease.

### Performance and comparison to benchmark

Generalization, i.e. the ability of a model to retain prediction performance on novel data, can be enhanced by partitioning the training sequences based on sequence identity. On the other hand, lowering the partitioning threshold too much eventually creates a reduced amount of clusters in which positives and negatives are intermixed, thus defeating the original purpose of homology partitioning and impairing the training process.

**Figure 4:**
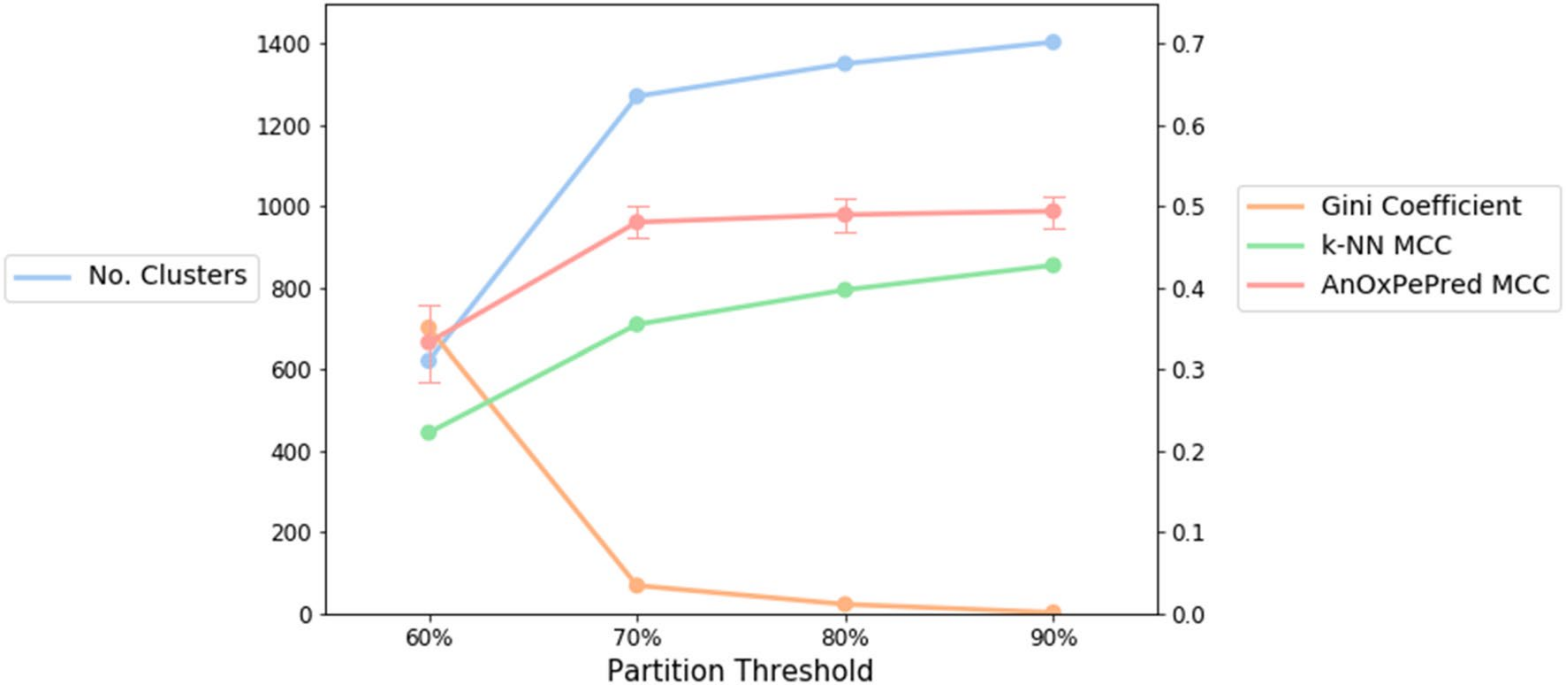
Overview of the effects of partitioning the data with different thresholds. Plotted is the performance of AnOxPePred and k-NN represented by the MCC of FRS, number of clusters (i.e. the number of groups of peptides based on the specified threshold) and the Gini coefficient based on the distribution of FRS peptides in each fold. *MCC* Matthew’s correlation coefficient, *FRS* free radical scavenger.

To identify the optimal similarity threshold, we analysed the number of clusters and the average Gini impurity index from co-clustered positives and negatives at different thresholds. This is displayed in Fig. 4 as a blue and orange line, respectively.

Additionally, the performance of k-NN and our model (represented by the MCC for FRS) are displayed as in green and red, respectively.

As seen in Fig. 4, the performance of k-NN decreases with a lower threshold, which is to be expected as it relies on sequence similarity. Meanwhile the performance of AnOxPePred is steady until 70%, and only decreases at 60% threshold. This implies that unlike the k-NN algorithm, AnOxPePred learns more general rules for predicting antioxidant activity than simple sequence similarity.

Additionally, a steep increase in Gini impurity and decrease in number of clusters occurs when going from a 70% to a 60% threshold. The latter is most likely caused by the high number of 3′mers which beneath 66% identity all ends up in one cluster prohibiting the creation of 5 even partitions.

**Figure 5:**
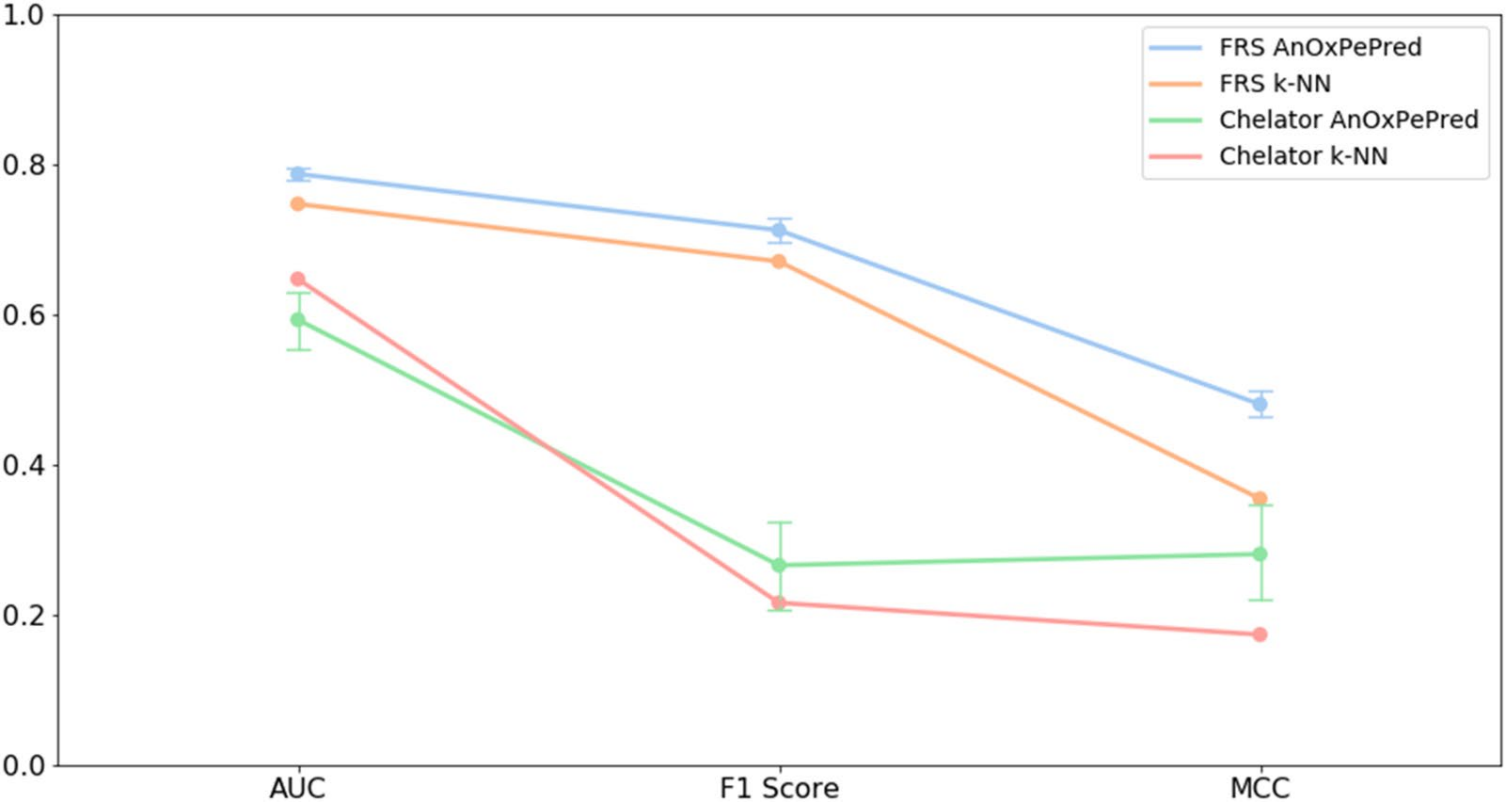
Performance comparison of AnOxPePred and k-NN for both FRS and chelating properties using the metrics AUC, F1 score and MCC. *AUC* area under the curve, *MCC* Matthew’s correlation coefficient, *FRS* free radical scavenger.

As the 70% threshold gives the partition with the lowest threshold while also retaining even partitioning, it was selected for evaluating and comparing the performances of the AnOxPePred and k-NN models. The models were trained and evaluated as described in methods. The resulting performances are compared in Fig. 5.

The performance of AnOxPePred is seen to outcompete the k-NN model in all metrics for FRS activity. For chelating activity AnOxPePred shows a better MCC and F1 score, but a lower AUC than k-NN. Additionally, it can be argued that AnOxPePred’s predictions are based on more general rules and not only the sequence similarity as the k-NN and thereby offer a more trustworthy prediction on a wider range of peptides. The better prediction performances for FRS, compared to chelating, are most likely caused by FRS data being more prevalent in the benchmark dataset. The exact metric values are shown in Supplementary Table S2.

### Experimental validation

In order to test the accuracy of our model on new peptides, and to understand the limits of our models, we measured the scavenging activity of 10 peptides with high predicted FRS scores from Patatin and Kunitz-Type proteinase inhibitors, which are proteins from potatoes that are abundant in byproducts of starch production. It is important to notice that two additional peptides that were initially selected could not be tested since they were not soluble in the reported experimental conditions. The results of the tests for all are reported in Table 2.

We can see that four peptides have scavenging activity as good or better than sodium caseinate, three have a comparable activity (an IC50 lower than twice the IC50 of sodium caseinate), and 3 have little scavenging activity. As most studies only isolate 1–10 antioxidant peptides from their protein sources, the high success rate of seven peptides out of ten with good scavenging activity demonstrates the accuracy and usefulness of the prediction^12,14,40,41^. On the other hand it is evident that especially for longer peptides, the tool in some cases fails, even if the peptide has a sequence composition similar to scavenging peptides in our benchmark set. Nonetheless, even a suboptimal prediction of larger peptides can be useful in the selection of possible antioxidants, as seen by our identification of a 22-residue antioxidant.

### Perspective

There is still plenty of room for further improvement of antioxidant peptide predictors, with the most pressing issue being the modest size of the benchmark dataset. Multi-task learning was used to draw information from small to long peptides and from FRS to chelating activity thereby improving their predictions. However, as larger peptides can form secondary structures, some crucial antioxidant information might not be obtained from short unstructured peptides. Further expansion of the current dataset to include a larger variety of peptides is therefore an important step for improvement of antioxidant peptide predictions, especially on peptides currently underrepresented in the dataset. Additionally, as our model relies on other features than the sequence, it is likely its performance would benefit more with an increased dataset than k-NN’s.

From the experimental validation we could see that, especially for the longer peptides tested, the prediction task is probably affected by more complex behaviours, such as local structure, that was not taken into account in the current algorithm. Additionally, some of the peptides we tested were not soluble. We believe that including peptide structure and solubility into the model can drastically improve its accuracy and usefulness.

Nonetheless, the results presented in this paper demonstrates the proof of concept of predicting antioxidant peptides based solely on their sequence, and that good predictions can be achieved. This indicates that AnOxPePred has the potential of becoming a useful tool by reducing the experimental work required when searching for new antioxidant peptides.

### AnOxPePred web-server

For the convenience of experimental scientists, the free web server, AnOxPePred (http://services.bioinformatics.dtu.dk/service.php?AnOxPePred-1.0), was developed. AnOxPePred is based on the model presented in this paper and will allow the user to predict the FRS and chelating properties of single peptides. In addition, another feature of AnOxPePred, is the ability to predict the activity of peptides within a protein. Here, the user can choose to predict the activity of all possible peptides, of length 2–30 amino acids, within the protein or the peptides that would be derived from hydrolysing the protein with a selection of conventional proteases. The input is given in a FASTA format and the output is a file with each peptide and their predicted antioxidant properties.

In summary, we introduce a deep convolutional neural network (CNN) classifier for predicting antioxidant activity of peptides, which illustrated a good performance and an ability to outperform a k-NN sequence identity-based approach, when predicting the free radical scavenging (FRS) and chelating properties of peptides.

Currently, an exhaustive and high-cost trial and error approach is used to identify antioxidant peptides. AnOxPePred is therefore not only a good benchmark for future antioxidant peptide predictors, but additionally, a useful computational tool to assist in the search of antioxidant peptides, thereby reducing the laboratory workload. To aid researchers identifying antioxidant peptides, the publicly accessible web-server, AnOxPePred (http://services.bioinformatics.dtu.dk/service.php?AnOxPePred-1.0), has been developed. Additionally, the source code is freely available at: (https://github.com/TobiasHeOl/AnOxPePred.git).

## Supplementary information

Supplementary Information.

## Data Availability

The dataset generated and used during this study is available at http://services.bioinformatics.dtu.dk/service.php?AnOxPePred-1.0 under the Dataset tab and included in this papers Supplementary Information files.
